# Effect of Ge Nanocrystals on 1.54 μm Photoluminescence Enhancement in Er_2_O_3_:ZnO and Ge Co-Sputtered Films

**DOI:** 10.3390/nano7100311

**Published:** 2017-10-11

**Authors:** Ranran Fan, Fei Lu, Kaikai Li

**Affiliations:** School of Information Science and Engineering, Shandong University, Jinan 250100, China; fanranran1@163.com (R.F.); likaikai_sdu@163.com (K.L.)

**Keywords:** photoluminescence, nanomaterials, rare-earth-doped materials, optical properties of thin films, deposition and fabrication

## Abstract

Photoluminescence (PL) of Er and Ge co-doped ZnO films synthesized by radio frequency magnetron co-sputtering was investigated. X-ray diffraction (XRD) patterns showed that the annealing process at 400–800 °C led to the formation of nanocrystal (nc) Ge. Samples containing nc-Ge showed a strong visible PL with a peak at 582–593 nm, which was consistent with the calculated energy of the exciton of the ~5 nm-sized nc-Ge, according to the quantum confinement effect. The formation of nc-Ge could greatly enhance the 1.54 μm emission, and it is considered that the 1.54 μm PL enhancement may come from a joint effect of both the energy transfer from nc-Ge to Er^3+^ and the local environment change of Er^3+^.

## 1. Introduction

Doping rare earth (RE) ion luminescence centers (such as erbium) into the carrier of semiconductors is an important method to fabricate light-emitting and light amplifier devices [1,2,3,4]. Efforts to enhance the visible and near-infrared luminescence of RE ions are on-going. It has been reported that oxygen co-doping and using wide band gap materials as the host material increased the Er luminescence intensity greatly [5,6]. Therefore, ZnO is believed to have become a favorable option to host Er luminescence centers in recent years, as it meets the condition of being an oxide and has a wide band gap of about 3.3 eV. In addition, the controllable electrical conductivity and the robust physics characteristics of ZnO is suitable for the fabrication of high-temperature resistant, high-frequency, and high-power optoelectronic devices [7,8,9,10]. 

However, the Er^3+^ luminescence in ZnO has not been satisfactory. In recent years, some experimental results show that nanocrystals could act as efficient RE sensitizers [11,12], and the luminescence efficiency of Er^3+^ could be greatly enhanced by the introduction of nanocrystals through the recombination of photogenerated exitons in them and the subsequent energy transfer to Er^3+^. The addition of Si nanocrystals (nc-Si) in Er-doped materials has been investigated extensively [13,14,15]. Especially, the observation of about two orders of magnitude enhancement of the room temperature photoluminescence (PL) from Er-doped silicon (Si)-rich Si oxide has generated great interest to develop small-sized and Si compatible optical amplifiers [16]. Various Si nanostructures have been shown to have much larger PL intensity for Er than that of SiO_2_ as a host. These nanostructures include porous Si, Si nanocrystals (nc-Si) in silica matrixes, and Si/SiO_2_ superlattice [17,18,19]. In contrast with nc-Si, Ge nanocrystals (nc-Ge) have many properties (wide size-dependent emission tunability, larger Bohr radius, etc.), making them superior to nc-Si [20,21]. Until now there have been several works on the synthesis of nc-Ge. Many methods, such as co-sputtering of Ge and SiO_2_, Ge ion implantation, and electron beam evaporation [22,23,24,25], have been employed to fabricate nc-Ge embedded in a host matrix (about 100–300 nm thick). Although there have been a limited number of reports about Ge-doped ZnO thin film structures [26,27], as far as we know no related research on the PL effect of Ge and Er co-doped ZnO (Ge:Er:ZnO) material has been carried out. In this paper, we investigate the PL properties of Ge:Er:ZnO film. X-ray diffraction (XRD) and transmission electron microscope (TEM) results prove that the diamond crystal of nc-Ge is formed by post-annealing. The 1.54 μm PL is greatly enhanced in a 600 °C annealed Ge:Er:ZnO sample, and it is considered that the PL enhancement may come from a joint effect of both the energy transfer from nc-Ge to Er^3+^ and the local environment change of Er^3+^. 

## 2. Materials and Methods

Ge:Er:ZnO films were prepared by co-sputtering Ge and ZnO:Er_2_O_3_ (Er ~0.06 at%) targets onto Al_2_O_3_ substrates. A ZnO:Er_2_O_3_ target of 7.6 cm diameter with several 5 mm× 5 mm Ge metal pieces were co-sputtered by radio frequency magnetron sputtering. In the current experiment, the Ge concentration was fixed to be about 4% in volume fraction. The deposition was carried out in an oxygen and argon mixed gas atmosphere (Ar/O_2_ = 2/5). The chamber pressure was maintained at a constant value of 0.7 Pa. After deposition, the film thickness was measured to be about 400 nm. Isochronal annealing of the films were performed for 20 min at temperatures from 400 up to 800 °C in N_2_ atmosphere. As comparison, the Er-doped ZnO (Er:ZnO) films without Ge were also deposited under the same experimental conditions. The crystallinity properties of the deposited films were characterized by X-ray diffraction (XRD). PL measurements were performed at room temperature using the 325 nm and 532 nm lasers to stimulate the visible and infrared PL signals, respectively, and a photomultiplier tube and thermo-electrically cooled InGaAs detector (DInGaAs1700-TE, Zolix, Beijing, China) were used to detect them.

## 3. Results and Discussion

**Figure 1:**
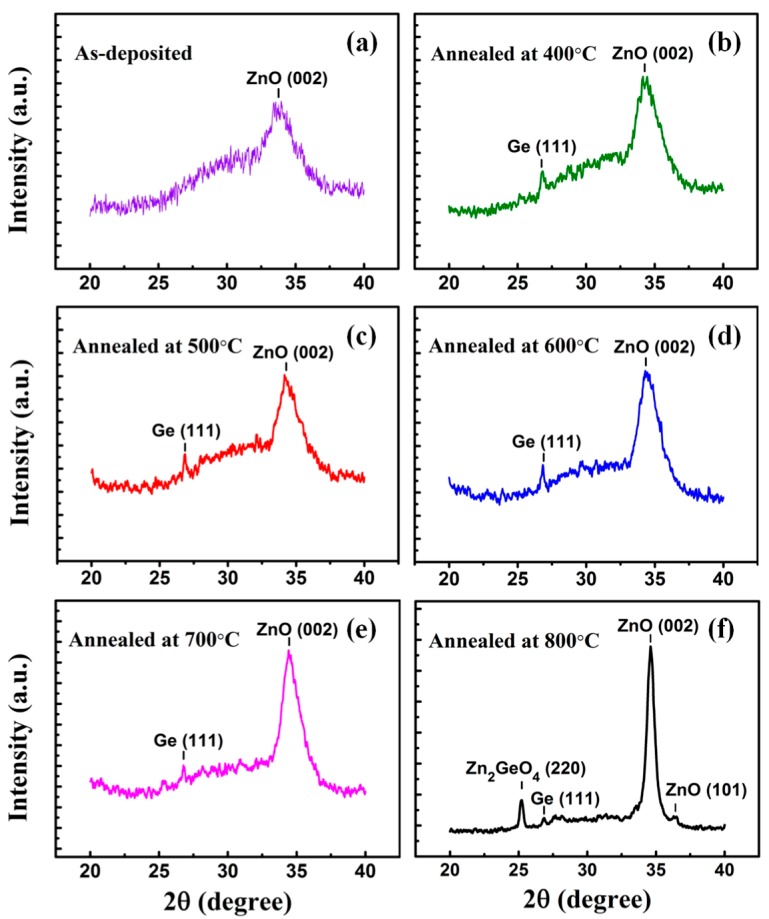
X-ray diffraction (XRD) patterns of (**a**) as-deposited Ge:Er:ZnO; Ge:Er:ZnO films annealed at (**b**) 400 °C; (**c**) 500 °C; (**d**) 600 °C; (**e**) 700 °C; (**f**) 800 °C.

Normalized diffraction XRD patterns of the as-deposited and annealed Ge:Er:ZnO films are demonstrated in Figure 1. A well-defined peak, identified as the (002) ZnO diffraction line, is clearly observed in the diagrams, indicating that the basal planes of the hexagonal ZnO structure are preferentially oriented parallel to the substrate surface. This oriented growth begins to be noticeable in as-deposited film, and is enhanced as annealing temperature increases, as it can be deduced from the evolution of the width and intensity of the corresponding peaks with temperature. The broad width of pattern in 400–700 °C annealed samples reveals that ZnO has a polycrystalline structure. A sharp (002) ZnO diffraction peak is detected in 800 °C annealed sample, indicating a better crystalline orientation of ZnO film. Meanwhile, new diffraction peaks from the Zn_2_GeO_4_ structure and (101) ZnO are also observed from this sample. The results show that high-temperature annealing can cause the interaction between ZnO and Ge, and subsequently form a new structure. It is remarkable that a noticeable (111) diffraction line from Ge face centered cubic (fcc) diamond lattice can be found in all the annealed samples. This diffraction peak from crystalline Ge is most evident in 600 °C sample and become no longer apparent in samples annealed at higher temperature. 

**Figure 2:**
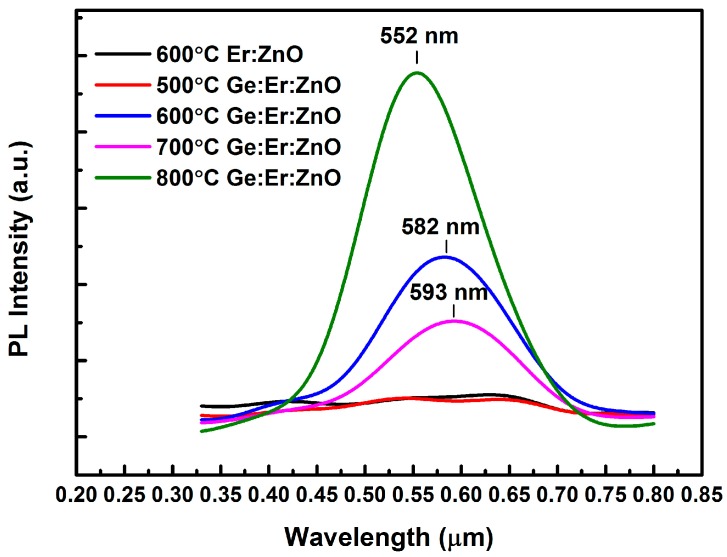
Visible photoluminescence (PL) spectra of the 600 °C annealed Er:ZnO film, and Ge:Er:ZnO films annealed at different temperatures.

Visible PL spectra from annealed films are shown in Figure 2. The broad spectra centered at 2.09, 2.13, and 2.25 eV are observed in these samples. It can be found that a PL peak appears in 600 °C annealed Ge:Er:ZnO sample and the intensity decreases after 700 °C annealing. Meanwhile the peak shifts from 582 to 593 nm. Several studies on nc-Ge-doped silica glasses have ascribed this intense PL around 2.2 eV to the existence of nanocrystalline Ge [28,29,30]. The red shift of the PL peak and broadening of the PL spectrum are attributed to the change of the average size of nc-Ge according to the quantum confinement effect. The results of 600 and 700 °C samples are consistent with this interpretation, and a detailed discussion is given later. The visible PL spectra may come from another emission source, that is, new defect states in ZnO originated from the incorporation of nc-Ge, as the defect-related band is clearly observed in a large set of undoped and doped ZnO layers [31]. It is shown in Figure 2 that a large PL intensity and blue shift of emission peak appears in 800 °C annealed sample. It can be found in Figure 1f that an obvious structural change occurs in this film. Not only the orientation of the ZnO structure becomes better, but also a new structure is formed. The visible PL signal in 800 °C annealed film should be primarily related to the ZnO matrix defects, because with the increase in nano-particle size under high temperature, the quantum effect of nc-Ge becomes negligible. 

To understand the quantum confinement effect in nc-Ge, Takagahara and Takeda proposed a theoretical explanation using the effective mass approximation and the Luttinger Hamiltonian. It was shown that a large red shift of the peak of PL spectra from visible to infrared region occurred as the nc-Ge diameter increased [32]. According to their calculation, the nc-Ge with a size of 2.1–2.3 nm contributed most to PL radiation in the energy range of 2.1–2.3 eV. The quantum confinement effect from nc-Ge became less pronounced when the dot radius was larger than 6 nm. This model provided a reasonable explanation for the visible PL of nc-Ge at ~2.2 eV, but the relation between the size of the nc-Ge and the energy range of PL was not supported by some experiments [33,34,35]. Many results showed that the shift of the peak energy of the PL spectrum was very small, or did not even depend on the average diameter of nc-Ge. Our experiments show that the energy of PL peak shifts only within a small range between 2.1 and 2.3 eV. It is similar as that of the above references, where the PL peak energy showed a constant energy at 2.0–2.1 eV when the sizes of nc-Ge were larger than 5 nm.

**Figure 3:**
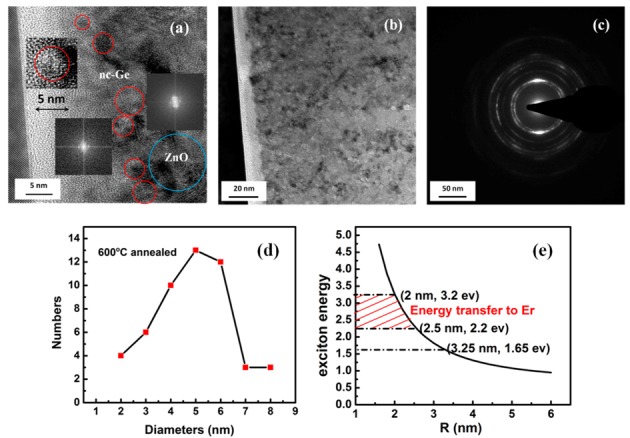
(**a**,**b**) High resolution transmission electron microscope (HRTEM) cross-section images of different proportional scale for the 600 °C annealed Ge:Er:ZnO film; (**c**) A diffraction pattern from the film; (**d**) Size distribution of nc-Ge in 600 °C annealed Ge:Er:ZnO film; (**e**) The relation of emission energy versus radius *R* of nc-Ge.

The Debye-Scherrer formula, *D* = 0.89λ*/B*cos*θ*, is firstly used to estimate the average size of the nc-Ge, where *D* is the diameter of the nc-Ge, λ is the wavelength of the X-ray, and *B* is the full width at half maximum (FWHM) of the X-ray diffraction peak at the diffraction angle (*θ*). The estimated diameter of nc-Ge for 600 °C and 700 °C samples correspond to 5.3 and 7.1 nm. The microstructure of the 600 °C sample is observed by high resolution transmission electron microscope (HRTEM) and the results are shown in Figure 3. From Figure 3a, it can be found that an amorphous ZnO layer grows near the substrate/Er:ZnO interface, then a polycrystalline Er:ZnO film with nc-Ge embedded in it is deposited. The FFT (fast Fourier transform) process is carried out to show the features of nc-Ge, which is shown in Figure 3a. The cubic lattice nc-Ge embedded in the hexagonal structure of the ZnO matrix can be clearly seen. The average size of the nc-Ge is about 5 nm, consistent with the estimation according to the XRD result. Figure 3d shows the size distribution of nc-Ge in the 600 °C annealed film, the majority of the nc-Ge is in the size of 4–6 nm. The calculated average size of them is about 4.9 nm, consistent with the results of the XRD and TEM. Several experiments have demonstrated that the visible nc-Ge PL phenomenon is consistent with the quantum confinement of electron-hole pairs between the widened band gap of nc-Ge. If Wannier excitons can be used to describe the quantum effect of nc-Ge [29], electron-hole pairs are supposed to be confined in an infinite spherical symmetry potential, the exciton Bohr radius $R_{B}$ is regarded as the critical size that distinguishes between two different situations of quantum confinement. Considering the actual size of the nc-Ge in the Er:ZnO film, a zeroth-order approximation asymptotic wave function of the exciton can be employed, where the electrons and holes are confined independently in the nc-Ge in radius *R*, and the energy can be given by:

(1) $$E_{nl}=E_{g}+\frac{\hbar^{2}}{2\mu_{e-h}}{\left(\frac{\alpha_{nl}}{R}\right)}^{2}-1.786\frac{e^{2}}{\varepsilon R}-0.248E_{R_{y}}^{*}$$

where *E_nl_* is the eigen value of energy; *E_g_* = 0.66 eV is a band-gap energy and $\alpha_{nl}$ is the eigen value of the zeroth-order spherical Bessel function; $\mu_{e-h}$ is the reduced mass for excitons, e is an electron charge, and ε is the static dielectric constant of Ge. Exciton binding energy $E_{R_{y}}^{*}$ is about 1–2 meV. *R* is the radius of nc-Ge, and the relation of emission energy versus radius *R* of nc-Ge is given in Figure 3b. Referring to the radius of nc-Ge obtained by TEM, *R* = 2.5 nm, the lowest energy *E*_10_ = 2.2 eV can be obtained using Equation (1). This value is consistent with our visible PL result.

**Figure 4:**
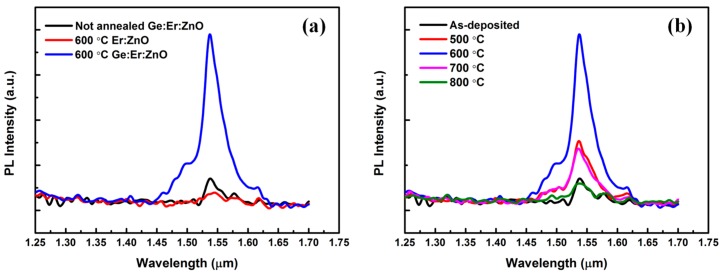
Infrared PL spectra of the (**a**) as-deposited Ge:Er:ZnO film, 600 °C annealed Er:ZnO and Ge:Er:ZnO films; (**b**) Ge:Er:ZnO films annealed at different temperatures.

To investigate the affection of Ge on Er infrared PL in Er:ZnO film, the PL spectra from the as-deposited, 600 °C annealed Ge:Er:ZnO, and 600 °C annealed Er:ZnO sample without Ge doping are measured. The result is shown in Figure 4a. It can be seen that the 1.54 μm emission of the samples without nc-Ge (as-deposited Ge:Er:ZnO and Er:ZnO films) are very low, indicating that the direct excitation of Er^3+^ is inefficient. However, this situation has been changed dramatically with the addition of nc-Ge, a strong Er^3+^ PL at 1.54 μm is observed from the 600 °C annealed Ge:Er:ZnO film. It provides an evidence for the enhancement effect of nc-Ge on 1.54 μm PL. This PL enhancement is closely related to the annealing conditions, as is shown in Figure 4b. When the sample is annealed from 400 °C to 800 °C, the 1.54 μm PL firstly increases with temperature to a maximum at 600 °C, and then decreases. 

**Figure 5:**
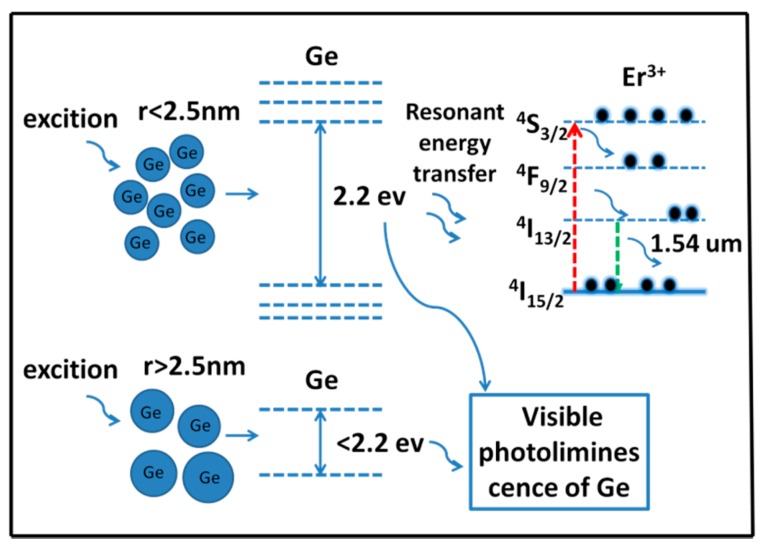
Schematic diagram for the proposed energy transfer process in the Ge:Er:ZnO film.

The PL enhancement of Er^3+^ may be related to the energy transfer from nc-Ge to Er^3+^. Figure 5 shows the schematic diagram for the possible energy transfer from nc-Ge to the surrounding Er^3+^ ions. Accroding to quantum confinement theory, when the quantum dot size is smaller than its effective Bohr radius (24 nm for Ge), with the decrease of the size, the carrier (electrons, holes) movement will be limited which leads to the increase of kinetic energy and the discrete of energy levels. Taking 600 °C sample as example, it shows the best 1.54 μm PL among all the samples. The size of ~5 nm nc-Ge dominates the 600 °C sample, the quantum confinement effect encourages excitation of nc-Ge at ~2.2 eV, and this energy is resonant with ^4^S_3/2_-^4^I_15/2_ of Er^3+^. When external excitation is applied, excited nc-Ge can either give rise to the visible emission itself, or resonantly transfer energy to the nearby Er^3+^ ions and causes an energy level transition of ^4^I_15/2_-^4^S_3/2_. The excited intra-4f electrons of Er^3+^ ions then jump down to the first excited level ^4^I_13/2_ through a non-radiative relaxation and give 1.54 μm emission by following the de-excitation transition to the ground level ^4^I_15/2_. A large absorption cross-section of nc-Ge and resonant energy transfer from nc-Ge to Er^3+^ could make the 1.54 μm emission more efficient. As is shown in Figure 3a, the radius of nc-Ge in the 600 °C sample is mostly in the range of 2–2.5 nm, which corresponds to the emission energy between 2.2 eV and 3.2 eV, according to the calculated result shown in Figure 3e. High-temperature treatment will cause the nc-Ge to grow to a larger size. When nc-Ge grows to be a larger cluster, the quantum confinement effect is less pronounced and energy transfer becomes weaker. This situation is consistent with the observation in the 600 °C and 700 °C samples. For the 800 °C sample, a decrease in the 1.54 μm PL may be due to two reasons: (1) the ZnO defect-related visible PL disperses partial energy of the direct 532 nm excitation, as high temperature induces the formation of larger Ge particles, which brings more grain boundaries and lattice dislocations in ZnO. New compounds, such as Zn_2_GeO_4_, also appear to act as defects in ZnO. All of them consume the excitation power; and (2) the decrease of the effective nc-Ge number, since high temperature not only promotes the formation of new compounds, but also part of Ge may exist in the ZnO film as Ge_2_O_3_ or Ge_2_O_3_-like precipitates. Both processes mean the depletion of nc-Ge. In addition to the energy transfer mechanism, changes in the local environment of Er^3+^ ions may also play an important role in luminescence enhancement. Our PLE spectra results show that, compared to the sample without Ge dopants, both the energy transfer from the ZnO host and direct excitation of Er^3+^ are more efficient in the nc-Ge-containing film, and the overall luminous efficiency is much higher in it. This indicates that the local environment of 4f states of Er ions has been modified by the formation of nc-Ge (1.54 μm photoluminescence enhancenment of Er^3^^+^-doped ZnO films containing Ge nanocrystals: joint effect from the Er^3+^ local environment changing and energy transfer of nc-Ge, Photonics Research, to be published). It has been reported that lowering of the symmetry of the crystal field around Er^3+^ is more suitable for Er intra-4f transitions [36]. The existence of nc-Ge may strongly affect the local environment of Er and enhances its transition rates. This could be another reason causing the 1.54 μm PL enhancement in Ge and Er co-doped ZnO.

## 4. Conclusions

In conclusion, 1.54 μm PL is enhanced in annealed Er:ZnO and Ge co-sputtered film. Nc-Ge is formed after annealing at a temperature above 400 °C. According to the exciton quantum model, visible PL at ~2.2 eV is mainly from the quantum confinement effect of nc-Ge at a size of 5–6 nm. It is found that the formation of nc-Ge could greatly enhance the 1.54 μm emission, the PL enhancement may come from a joint effect of both the energy transfer from nc-Ge to Er^3+^ and the local environment change of Er^3+^.

## References

[1] Douglas L., Mundle R., Konda R., Bonner C.E., Pradhan A.K., Sahu D.R., Huang J. (2008). Influence of doping rate in Er^3+^: ZnO films on emission characteristics. Opt. Lett..

[2] Jang Y.R., Yoo K.H., Ahn J.S., Kim C., Park S.M. (2011). 1.54 μm emission mechanism of Er-doped zinc oxide thin films. Appl. Surf. Sci..

[3] Bubendorff J.L., Ebothé J., el Hichou A., Dounia R., Addou M. (2006). Luminescent spectroscopy and imaging of textured sprayed Er-doped ZnO films in the near ultraviolet and visible regions. J. Appl. Phys..

[4] Conti G.N., Chiasera A., Brenci M., Ferrari M., Pelli S., Sebastiani S., Tosello C., Righini G.C. (2006). Er^3+^/Yb^3+^-codoped silica-germania sputtered films: Structural and spectroscopic characterization. J. Non-Cryst. Solids.

[5] Takahei K., Taguchi A. (1993). Selective formation of an efficient Er-O luminescence center in GaAs by metalorganic chemical vapor deposition under an atmosphere containing oxygen. J. Appl. Phys..

[6] Ishii M. (2001). Local structure analysis of an optically active center in Er-doped ZnO thin film. J. Appl. Phys..

[7] Ko Y.H., Lee S.H., Yu J.S. (2013). Performance enhanced piezoelectric ZnO nanogenerators with highly rough Au electrode surfaces on ZnO submicrorod arrays. Appl. Phys. Lett..

[8] Trioloa C., Fazio E., Neri F., Mezzasalma A.M., Trusso S., Patanè S. (2015). Correlation between structural and electrical properties of PLD prepared ZnO thin films used as a photodetector material. Appl. Surf. Sci..

[9] Ahn H., Kao N., Liu W., Hsieh W. (2017). THz Study on the Role of ZnO Crystallinity in ZnO/AgNW/ZnO Composite Electrodes. IEEE J. Sel. Top. Quantum Electron..

[10] Yin Z., Liu X., Wang H., Wu Y., Hao X., Ji Z., Xu X. (2013). Light transmission enhancement from hybrid ZnO micro-mesh and nanorod arrays with application to GaN-based light-emitting diodes. Opt. Express.

[11] Chiasera A., Macchi C., Mariazzi S., Valligatla S., Lunelli L., Pederzolli C., Rao D.N., Somoza A., Brusa R.S., Ferrari M. (2013). CO_2_ Laser irradiation of GeO_2_ planar waveguide fabricated by rf-sputtering. Opt. Mater. Express.

[12] Zur L., Tran L.T.N., Meneghetti M., Tran V.T., Lukowiak A., Chiasera A., Zonta D., Ferrari M., Righini G.C. (2017). Tin-dioxide nanocrystals as Er^3+^ luminescence sensitizers: Formation of glass-ceramic thin films and their characterization. Opt. Mater..

[13] Kik P.G., Brongersma M.L., Polman A. (2000). Strong exciton-erbium coupling in Si nanocrystal-doped SiO_2_. Appl. Phys. Lett..

[14] Izeddin I., Timmerman D., Gregorkiewicz T. (2008). Energy transfer in Er-doped SiO_2_ sensitized with Si nanocrystals. Phys. Rev. B.

[15] Lu Y., Huang C., Cheng J., Larsen A.N. (2016). High Er^3+^ luminescent efficiency in Er-doped SiO*x* films containing amorphous Si nanodots. J. Alloys Compd..

[16] Kenyon A.J., Trwoga P.F., Federighi M., Pitt C.W. (1994). Optical properties of PECVD erbium-doped silicon-rich silica: Evidence for energy transfer between silicon microcIusters and erbium ions. J. Phys. Condens. Matter.

[17] Henley W., Koshka Y., Lagowski J., Siejka J. (2000). Infrared photoluminescence from Er doped porous Si. J. Appl. Phys..

[18] Kenyon A.J., Chryssou C.E., Pitt C.W., Shimizu-Iwayama T., Hole D.E., Sharma N., Humphreys C.J. (2002). Luminescence from erbium-doped silicon nanocrystals in silica: Excitation mechanisms. J. Appl. Phys..

[19] Timoshenko V.Y., Lisachenko M.G., Kamenev B.V., Shalygina O.A., Kashkarov P.K., Heitmann J., Schmidt M., Zacharias M. (2004). Highly efficient sensitizing of erbium ion luminescence in size-controlled nanocrystalline Si/SiO_2_ superlattice structures. Appl. Phys. Lett..

[20] Takeoka S., Fujii M., Hayashi S., Yamamoto K. (1998). Size-dependent near-infrared photoluminescence from Ge nanocrystals embedded in SiO_2_ matrices. Phys. Rev. B.

[21] Wang Z., Wang S., Yin Y., Liu T., Lin D., Li D., Yang X., Jiang Z., Zhong Z. (2017). Promising features of low-temperature grown Ge nanostructures on Si (001) substrates. Nanotechnology.

[22] Skorupa W., Rebohle L., Gebel T. (2003). Group-IV nanocluster formation by ion-beam synthesis. Appl. Phys. A.

[23] Choi W.K., Ho V., Ng V., Ho Y.W., Ng S.P., Chim W.K. (2005). Germanium diffusion and nanocrystal formation in silicon oxide on silicon substrate under rapid thermal annealing. Appl. Phys. Lett..

[24] Avella M., Prietoa A.C., Jimemenez J., Rodriguez A., Sangrador J., Rodrguez T. (2005). Violet luminescence in Ge nanocrystals/Ge oxide structures formed by dry oxidation of polycrystalline SiGe. Solid State Commun..

[25] Agan S., Celik-Aktas A., Zuo J.M., Dana A., Aydinli A. (2006). Synthesis and size differentiation of Ge nanocrystals in amorphous SiO_2_. Appl. Phys. A.

[26] Shih G.H., Allen C.G., Potter B.G. (2012). Interfacial effects on the optical behavior of Ge:ITO and Ge:ZnO nanocomposite films. Nanotechnology.

[27] Ceylan A., Ali J.M., Ozcan S. (2013). Synthesis of ZnO: Ge nanocomposite thin films by plasma gas condensation. Mater. Sci. Semicond. Process..

[28] Maeda Y., Tsukamoto N., Yazawa Y., Kanemitsu Y., Masumoto Y. (1991). Visible photoluminescence of Ge microcrystals embedded in SiO_2_ glassy matrices. Appl. Phys. Lett..

[29] Maeda Y. (1995). Visible photoluminescence from nanocrystallite Ge embedded in a glassy SiO_2_ matrix: Evidence in support of the quantum-confinement mechanism. Phys. Rev. B.

[30] Venkatasubramanian R., Malta D.P., Timmons M.L., Hutchby J.A. (1991). Visible light emission from quantized planar Ge structures. Appl. Phys. Lett..

[31] Reshchikov M.A., Morkoc H., Nemeth B., Nause J., Xie J., Hertog B., Osinsky A. (2007). Luminescence properties of defects in ZnO. Phys. B.

[32] Takagahara T., Takeda K. (1992). Theory of the quantum confinement effect on excitons in quantum dots of indirect-gap materials. Phys. Rev. B.

[33] Kanemitsu Y., Uto H., Masumoto Y., Maeda Y. (1992). On the origin of visible photoluminescence in nanometer-size Ge crystallites. Appl. Phys. Lett..

[34] Okamoto S., Kanemitus Y. (1996). Photoluminescence properties of surface-oxidized Ge nanocrystals: Surface localization of excitons. Phys. Rev. B.

[35] Paine D.C., Caragianis C., Kim T.Y., Shigesato Y., Ishahara T. (1993). Visible photoluminescence from nanocrystalline Ge formed by H_2_ reduction of Si_0.6_Ge_0.4_O_2_. Appl. Phys. Lett..

[36] Zhou Z., Komori T., Ayukawa T., Yukawa H., Morinaga M., Koizumi A., Takeda Y. (2005). Li- and Er-codoped ZnO with enhanced 1.54 μm photoemission. Appl. Phys. Lett..

